# Bifurcation-based embodied logic and autonomous actuation

**DOI:** 10.1038/s41467-018-08055-3

**Published:** 2019-01-10

**Authors:** Yijie Jiang, Lucia M. Korpas, Jordan R. Raney

**Affiliations:** Department of Mechanical Engineering and Applied Mechanics, 220 S 33rd St., University of Pennsylvania, Philadelphia, PA 19104 USA

## Abstract

Many plants autonomously change morphology and function in response to environmental stimuli or sequences of stimuli. In contrast with the electronically-integrated sensors, actuators, and microprocessors in traditional mechatronic systems, natural systems embody these sensing, actuation, and control functions within their compositional and structural features. Inspired by nature, we embody logic in autonomous systems to enable them to respond to multiple stimuli. Using 3D printable fibrous composites, we fabricate structures with geometries near bifurcation points associated with a transition between bistability and monostability. When suitable stimuli are present, the materials swell anisotropically. This forces a key geometric parameter to pass through a bifurcation, triggering rapid and large-amplitude self-actuation. The actuation time can be programmed by varying structural parameters (from 0.6 to 108 s for millimeter-scale structures). We demonstrate this bioinspired control strategy with examples that respond to their environment according to their embodied logic, without electronics, external control, or tethering.

## Abstract (web summary)

The demonstration of nature-inspired embodied control logic in simple autonomous material systems is of significant interest in applications such as soft robots and drug delivery. Here, the authors demonstrate 3D printed self-actuating structures with control logic in response to suitable stimuli.

## Introduction

Responsiveness to environmental stimuli is vital to the function and growth of plant life^1–6^. Natural morphological changes can take hours (e.g., for dispersal of seeds or environmental adaptation^3–5^) or less than a second (e.g., the closing of the Venus flytrap, *Dionaea muscipula*, to capture prey^6,7^). These can also require multiple stimuli or a specific sequence of stimuli. For example, underlying the motion of the Venus flytrap is a complex sequence of sensing and actuation events. Hairs on the leaf lobes must be mechanically stimulated multiple times within 20–30 s for the lobes to close. After partially closing, the plant reassesses the size of any enclosed object and awaits continued stimulation to determine whether to open (to release the object) or to close the rest of the way (to begin digestion)^7,8^. In traditional mechatronic systems, sensing, actuation, and control are performed electronically. Complex natural logic, however, is embodied in plants more directly through the tools nature has at its disposal: stimuli-induced morphological changes via material composition and the structural (geometric) organization. Recent work in soft robotics has shown the feasibility of embedding control logic in the structure of the robot itself (i.e., microfluid logic)^9^, opening the door for further nature-inspired control systems, as we explore here.

The ability of a material to adapt to its environment via response to specific stimuli is of interest for numerous applications, from robotics^10–12^ to medicine (e.g., drug delivery^13,14^). Synthetic active materials have been developed that actuate in response to a variety of stimuli^15–23^, including chemical cues^24^, temperature^10,11,25–31^, light^12,32–36^, voltage^37–39^, and water^40–42^. Each of these stimuli-responsive materials has its own set of strengths as well as practical challenges that need to be addressed for its use in autonomous applications. For example, thermally-activated shape changes, such as those associated with shape-memory polymers^11,25,27,43,44^, often require mechanical programming and/or large thermal energy transfer to/from the environment. Light- or voltage-responsive materials often require a stimulus of significantly larger magnitude than may be encountered in the ambient environment (e.g., high illumination intensity^33,45^ or high voltage^37,38^). Actuation via differential swelling (e.g., using bilayers^42,46,47^ or anisotropic composite materials^40^) requires diffusion of stimuli (e.g., solvents), which, depending on sample volume and geometry, can take many minutes due to the intrinsic limits of the speed of diffusion^40,41^. Moreover, in these examples, the material strain that is induced is a monotonic function of the amount of stimulus that is present, rather than a discrete, sudden morphological change.

The active materials above achieve their responsiveness via composition and microstructure, i.e., via the functionality intrinsic to the specific polymer matrix or (if a composite) to microstructural features, such as fiber alignment. However, the *geometric* arrangement of the material is also vital to the overall actuation response. Natural systems like plants possess the same speed limits associated with diffusion that synthetic materials do^1^. When faster responses are required, however, plants increase the rate of actuation using geometrically defined instabilities^6^, a nonlinear mechanical phenomenon studied extensively^48–50^ in structures, such as beams^51–57^ and shells^29,46,58^. Snap-through instabilities are associated with rapid morphological changes. These have been used in previous work to enhance the speed of actuation in soft materials, including in response to diffusion. Examples include diffusion-induced actuation via doubly curved beams and multilayer shells with stiffness gradients^46,57,58^, and structures composed of layers with orthogonal microstructure^29^.

In this work, we combine anisotropic materials with bistable structures to embody logic (AND, OR, and NAND) in 3D printable composite structures. These structures can be programmed to respond to multiple stimuli and to actuate at specific times (and in sequences of defined actuation events). We accomplish this by 3D printing self-actuating units consisting of laterally-constrained fibrous composite beams. We design the beams to possess geometries near bifurcation points that govern their stability behavior. The beams swell anisotropically in the presence of suitable stimuli, causing a key geometric parameter to move through a bifurcation point. This triggers rapid and large-amplitude actuation at pre-defined times and in response to multiple stimuli. Specifically, we use *polydimethylsiloxane* (PDMS)-based and *hydrogel*-based materials to respond to non-polar solvents and water, respectively.

## Results

### General concept

**Fig. 1:**
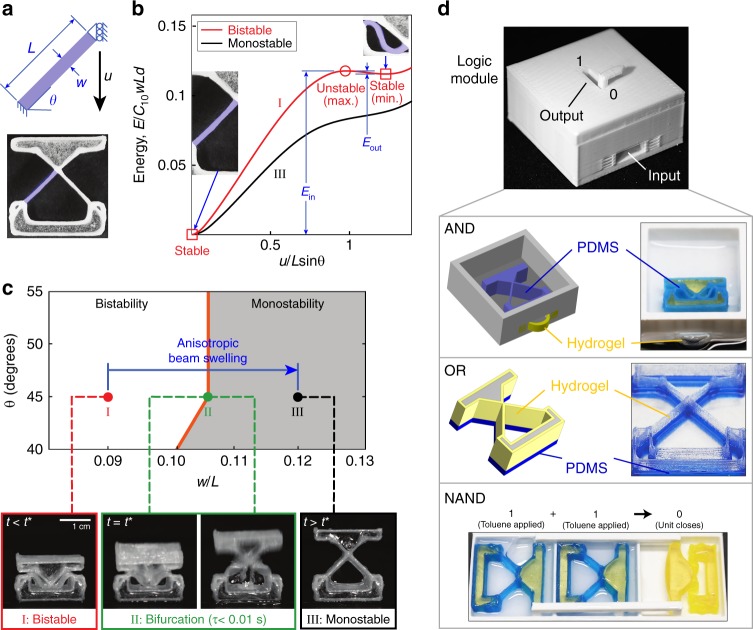
Overview of bifurcation-based actuation. **a** Schematic and photo of beams, with key geometric parameters and boundary conditions. **b** The normalized strain energy, *E*/(*C*_10_*wLd*), where *C*_10_ is a material parameter in the Holzapfel–Gasser–Ogden (HGO) model^65^ and *d* is the out-of-plane thickness of the beam; plots are included for a bistable beam (red curve, I) and a monostable beam (black curve, III) as a function of normalized displacement, *u/Lsinθ*, obtained via finite element simulations (Supplementary Note 5). Inset: photos of the undeformed (*u* = 0) and buckled configurations, corresponding to the two stable points (energy minima) on the bistable curve; the monostable curve has only the single minimum at *u* = 0. **c** Geometric phase diagram mapping geometric parameters to mechanical behavior, with schematic overlay indicating the transition from bistable to monostable (point I to point III) due to anisotropic swelling of the beams. Also shown are representative images recorded with a high-speed camera during actuation showing a unit when it is bistable (red), at the point of bifurcation when actuation occurs (green), and monostable (black). **d** A logic module which can act as one of several types of logic gates depending on the contents of the module. An AND gate can be constructed by using a hydrogel valve and a PDMS-GF15 bistable unit (requiring both water and toluene to switch from 0 to 1). An OR gate can be constructed by fabricating a composite bistable unit from both PDMS-GF5 (blue) and hydrogel (transparent) materials (this actuates if either water or toluene is applied); a functionally-complete NAND gate can be constructed by connecting two “input” bistable units to one “output” unit. In this case, only when toluene is applied to both input units will the output unit close from 1 to 0 (i.e., 1+1→0)

Laterally-constrained beams, depending on their geometric parameters and boundary conditions, can exhibit very different mechanical responses, including cantilever-like bending, snap-through instabilities^53^, and bistability^51,52,59^. Each of these behaviors correspond to specific domains in a parameter space defined by beam geometry. For laterally-confined, tilted beams, these key geometric parameters are the inclination angle of the beam (*θ*) and its slenderness ratio (*w/L*, where *w* denotes the beam width and *L* denotes the beam length) (Fig. 1a). We used finite element simulations (discussed in detail later) to obtain the bistable and monostable energy curves in Fig. 1b (curves I and III, respectively), both with *θ* = 45^o^ but with different *w/L* ratios (corresponding to points I and III in Fig. 1c). A bistable mechanical response is defined by a dual-well potential (Fig. 1b), with each well representing a stable morphology that can be maintained without any applied force or continued input of energy^51,52^. The two stable configurations are separated by an energy barrier that, if traversed, produces an instability and a rapid snap-through from one stable configuration to the other. The two stable wells are not at the same energy level because in one configuration the beam is unstrained, and in the other it is buckled (associated with strain energy). The size of the energy barrier, i.e., the depth of the second well (*E*_*out*_ in Fig. 1b), can be controlled by the geometry of the beams^51,56,59^. For a fixed *θ*, as *w*/*L* increases (i.e., as the beam becomes wider relative to its length) the energy barrier decreases, reaching a value of zero at a specific value of *w*/*L* that defines a bifurcation point, *B* (at point II in Supplementary Figure 1). For values of *w*/*L* larger than *B*, the beam is monostable, meaning it will return to its undeformed position (*u* = 0) if the applied force is removed. The critical value of *w*/*L* = *B* changes as a function of *θ*, as represented by the orange line dividing bistable from monostable regions in Fig. 1c.

Because of this sharp critical value, only a small nudge to the geometric parameters (e.g., via active materials) is necessary to cross a bifurcation point and trigger rapid snap-through for the purpose of actuation or movement. To accomplish this, we fabricate the beams using highly anisotropic composite materials, causing anisotropic swelling of the beams upon exposure to material-specific cues (e.g., water or non-polar solvents in our initial work). As shown in Fig. 1b and c, for a beam fabricated with geometry corresponding to point I (*w*/*L* < *B*) and placed in its buckled configuration, if it were to anisotropically swell to point III (*w*/*L* > *B*) it would have to pass through the bifurcation point at point II (*w*/*L*=*B*=0.105), above which the beam is only stable in its unbuckled configuration (*u* = 0). This forces a rapid actuation event, rapidly releasing the strain energy from the beam as it ceases to be bistable. Supplementary Movie 1 shows such an actuation event, recorded using a high-speed camera during bifurcation (in this case, PDMS-glass fiber composite beams responding to toluene). The images in Fig. 1c demonstrate the rapid actuation (*τ* < 0.01 s) that occurs for any beam that traverses such a bifurcation point. *τ* represents the amount of time it takes for the actuation event (the release of the stored strain energy) once bifurcation is reached (as observed with a high-speed camera). Note, the value of *w/L* would remain unchanged during swelling of an isotropic beam, and thus the bifurcation point would not be reached horizontally. (Depending on the specific architecture, isotropic swelling could still lead to the crossing of a bifurcation point via an increase in *θ*. However, since the monostable/bistable boundary is nearly vertical, it would typically require a larger degree of material swelling to do so.) Supplementary Movies 1–3 demonstrate the feasibility of using changes to the environment to trigger snap-through instabilities^35,44,46,60^, as also observed in nature^1^. For example, Supplementary Movie 3 shows a hopper that autonomously jumps out of the way when an undesired chemical (in this case, toluene) enters its environment. We note that this behavior occurs without discrete sensors, tethering, or mechatronic actuators: the system’s combination of composition and structure is itself the sensor, control system, and actuator. By harnessing systems of 3D printable bistable, anisotropic beam units (each of which can be independently assigned its own choice of actuation timing and its own stimulus, as described later), our bioinspired approach allows the “embodiment” of complex control in this material-structure combination.

In Fig. 1d, we demonstrate modular embodied logic, in which a mechanical logic module can produce AND, OR, or NAND output in response to chemical inputs, depending on which structures are placed inside the module. We produce an AND gate by using a hydrogel valve and a PDMS-GF15 bistable unit inside (Supplementary Figure 2). The actuation of this inner bistable unit flips the output of the module from 0 to 1. However, to achieve this, water must be present (to make the hydrogel swell and buckle, opening the valve) as well as toluene (to trigger the actuation of the unit). We produce an OR gate by constructing a bistable unit with out-of-plane arrangements of both PDMS-GF5 (blue) and hydrogel (transparent) materials. This bistable unit can actuate when either water or toluene is applied (Supplementary Movie 4). Finally, we produce a NAND gate by connecting two units (corresponding to two inputs) and one output. The stiffness of these is tuned (via geometry) such that both inputs must actuate in order to close the output unit (i.e., 1+1→0). Otherwise, the output unit remains open (Supplementary Movie 5). Since NAND is a functionally complete logic gate, in principle any gated logic could be achieved via combinations of these. To chain the logic gates into more complex systems, the mechanical response from one gate (the output) could be used either to indicate when to perform a manual pour, or to automatically open a chamber to allow fluid movement to the next gate (e.g., the input solvents for the NAND gate could be either manually poured or introduced automatically via suitable channel design).

### Materials and fabrication

In the remainder of this article, we outline how to achieve this behavior, and demonstrate utility via several use cases.

To achieve the general strategy outlined above, the ideal materials would meet the following requirements: First, each material must swell in response to a defined stimulus, e.g., water, non-polar solvents, temperature, light, etc. (in this work, we demonstrate the use of the first two of these stimuli, but any would work). Second, the materials swell *anisotropically* to alter the key geometric parameter (*w/L*) when exposed to their stimuli, allowing a well-controlled approach to move through a bifurcation. Third, the materials should be sufficiently elastic to maintain a buckled configuration (maximum material strain of *ε*_max_ < 0.15) without prohibitive time-dependent relaxation, as it is this stored elastic energy that enables the actuation event. Finally, the materials (or material precursors) must be patternable with high structural fidelity, since the mechanical response of the beams is determined by precise values of geometric parameters.

**Fig. 2:**
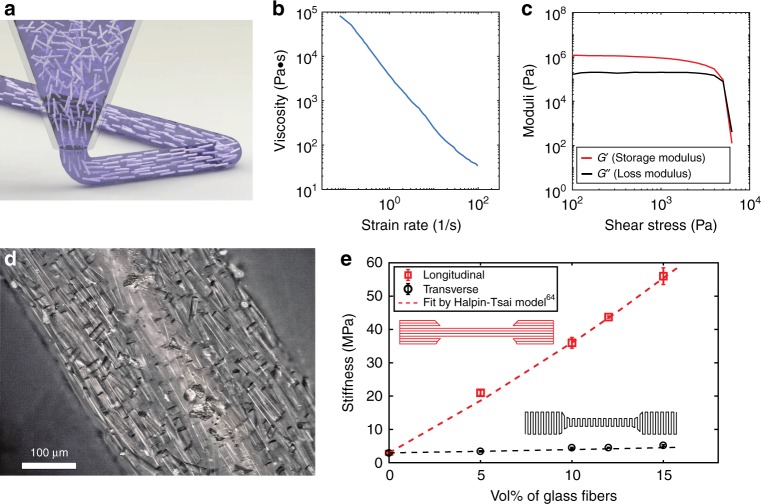
Direct ink writing (DIW) of PDMS-glass fiber composites. **a** Schematic of extrusion of PDMS ink during DIW 3D printing, leading to alignment of glass fibers. **b**, **c** Rheological characterization of the PDMS-GF ink shows shear-thinning and yield-stress behavior, respectively. **d** An optical micrograph showing material printed using PDMS-GF ink (15 vol% glass fibers), with alignment of glass fibers along the print direction. **e** Stiffness of PDMS-GF composites (after curing of printed structures) as a function of the volume fraction of glass fibers and the fiber orientation, based on tensile testing of printed specimens (“Longitudinal” and “Transverse” indicate printing and fiber orientation that is parallel with or perpendicular to the loading direction, respectively), and fit using the Halpin–Tsai model^64^

While 3D printers make it relatively easy to achieve sufficient structural fidelity of the beams (the fourth point above), most conventional 3D printers are unable to simultaneously achieve the other requirements^61^. Direct ink writing (DIW) is an extrusion-based 3D printing technique^61,62^ which offers a large degree of materials flexibility, and is ideal for producing anisotropic materials (fibers align due to material shear in the nozzle^40,63^ (Fig. 2a)). This approach allows us to meet all four criteria above, but requires some rheological tuning of the materials to allow printing (Fig. 2b–d and Supplementary Figure 3). In this work, we make use of two materials that respond to two distinct stimuli: a PDMS-based material (containing short glass fibers to provide anisotropy), which swells in the presence of non-polar solvents, such as toluene or hexane; and a hydrogel-based matrix (containing cellulose fibrils to provide anisotropy) that swells in the presence of water. Our PDMS ink formulation (see Methods) shows the rheological properties desired for DIW, including a decrease in apparent viscosity with increasing shear rate (Fig. 2b) and a viscoelastic yielding behavior that is characterized by a high storage modulus (G') when the shear stress is low (allowing the material to maintain its shape and to behave like an elastic solid) and a defined yield stress above which the storage modulus suddenly drops (allowing flowability through the nozzle) (Fig. 2c). A similar rheological profile is observed for the hydrogel material (Supplementary Figure 3). Because of the alignment of the fibers during extrusion (Fig. 2d), a high degree of mechanical anisotropy can be achieved. We characterize this by printing tensile specimens with fiber alignment both parallel with (“Longitudinal”) and perpendicular to (“Transverse”) the loading direction, subsequently testing these under quasistatic tension (see Methods) (Fig. 2e). Since the glass fibers are much shorter than the beam length, the Halpin–Tsai model^64^ is used, with the best fit corresponding to a matrix stiffness of *E*_m_ = 2.96 MPa and a fiber stiffness of *E*_f_ = 52.13 GPa (details in Supplementary Note 3). With increasing glass fiber volume fraction, the longitudinal stiffness, *E*_L_, increases much more than the transverse value, *E*_T_ (Fig. 2e), as expected for short fiber composites. With 15 vol% glass fibers, significant mechanical anisotropy (*E*_L_*/E*_T_ = 10.8) is obtained while good printability is maintained. Additional tensile tests on swollen samples and stress relaxation tests are shown in Supplementary Figure 4.

### Mechanical behavior

**Fig. 3:**
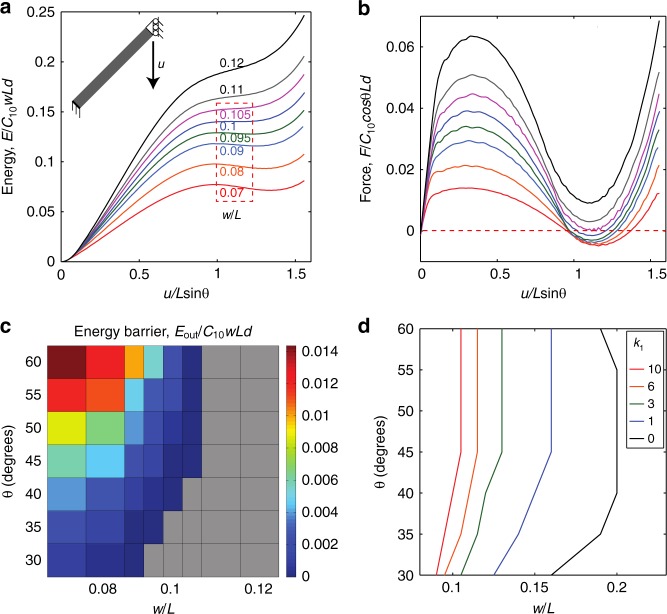
Finite element analysis to determine the phase boundary for anisotropic materials. **a**, **b** Energy and force, respectively, as a function of displacement, for a beam with *θ* = 45^o^ and a *w/L* ratio varying from 0.07 to 0.12. The dashed box indicates bistable *w/L* values. **c** Energy barrier, *E*_out_, necessary for a buckled beam to snap back to its undeformed configuration (the gray region indicates that the beam is monostable). **d** The phase boundaries as a function of anisotropy, where *k*_1_=0 indicates an isotropic material and increasing *k*_1_ indicates increasing anisotropy (i.e., larger stiffness ratio *E*_L_/*E*_T_); everything to the left of a particular line is bistable for that value of *k*_1_

In prior work on the bistability of laterally-constrained beams^51^, the relationship between beam geometry (the two key geometric parameters *θ* and *w*/*L*) and the stability behavior (e.g., bistable or monostable) was considered material-independent. However, this conclusion implicitly assumed that the material was isotropic. In fact, the degree of material anisotropy is also essential for determining the stability. Using experiments and finite element analysis (FEA), we locate the boundary between regions of monostability and bistability in the geometric phase diagram (e.g., Fig. 1c) for the more general case, in which the material anisotropy is allowed to vary. We first fit an anisotropic hyperelastic mechanical model, the Holzapfel–Gasser–Ogden (HGO) model^65^, to our experimental tensile data for the case of 15 vol% glass fibers (Supplementary Figure 5a) and then use this to conduct a parametric study (Fig. 3). The results (Fig. 3a, b) indicate that a beam with *θ* = 45° is bistable if *w/L* is less than 0.105 (i.e., the second energy well exists, and therefore the quantity *E*_*out*_ can be defined and is nonzero), and monostable otherwise (Supplementary Note 5). This is consistent with our experimentally-determined phase boundary between 0.102 < *w/L* < 0.108 (Supplementary Note 4). We develop a more complete phase diagram by systematically varying *θ* and *w/L* and determining the normalized energy barrier *E*_out_/(*C*_10_*wLd*) (Fig. 3c), which is very different from the isotropic case^51^. As discussed more in Supplementary Note 5, the phase boundary depends strongly on the degree of anisotropy (see Fig. 3d and Supplementary Figure 5c, with *k*_1_ = 0 indicating isotropy and increasing *k*_1_ indicating increasing anisotropy). For a beam of fixed length, *L*, the greater the degree of material anisotropy, the thinner the beam must be to be bistable (Supplementary Note 5).

**Fig. 4:**
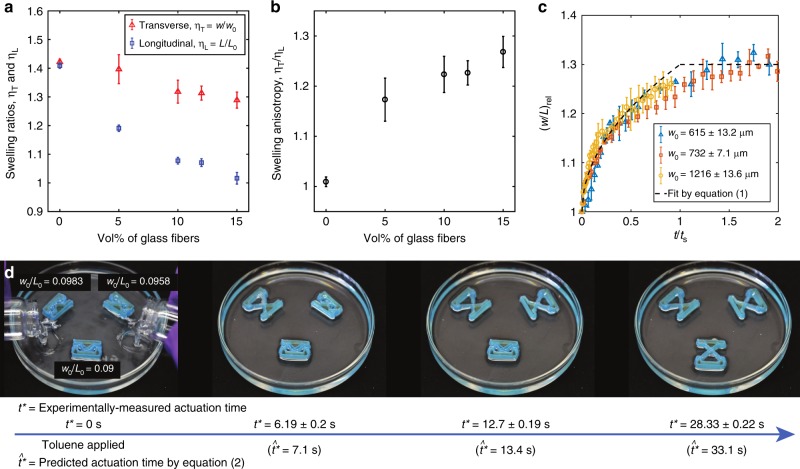
Control of actuation time. **a** Swelling ratio in the transverse (*η*_T_ = *w/w*_0_) and longitudinal (*η*_L_ = *L*/*L*_0_) directions for PDMS–GF composite materials after submersion in toluene for one day. **b** Swelling anisotropy, *η*_T/_*η*_L_ as a function of volume fraction of glass fibers. **c** The relative slenderness ratio (*w/L*)_rel_ as a function of time for beams with different initial widths (time is normalized by saturation time, *t*_s_, to account for the dependence of diffusion time on volume of material). **d** Actuation time can be controlled by selecting specific values for the initial beam geometry, *w*_0_/*L*_0_. The measured time t^*^ is given, as well as the predicted time, $\hat t^ \ast$, as calculated from equation (2). The error bars are the standard deviations computed from multiple measurements

The anisotropy in stiffness imparted by the alignment of the glass fibers produces anisotropic swelling when the material is exposed to a suitable solvent (Fig. 4). To characterize this, we measured *L* and *w* of the printed beams during exposure to the solvent under an optical microscope. Since PDMS swells strongly in non-polar solvents, we used toluene as the stimulus^66^. When allowed to become fully saturated by toluene (at time *t* = *t*_s_), the isotropic PDMS matrix (no glass fibers) swells until each linear dimension has increased by 42%, corresponding to a swelling ratio of *η* = 1.42 (see Fig. 4a and Supplementary Note 6). The mechanical anisotropy produced by aligned fibers (e.g., in PDMS-GF composites) significantly reduces the swelling ratio in the direction of fiber alignment (longitudinal direction), *η*_L_ = *L*_*s*_/*L*_0_. The magnitude of this reduction increases as the volume fraction of the fibers (and concomitantly the degree of anisotropy) increases. In contrast, there is only a slight reduction in the swelling ratio in the direction perpendicular to the fibers (the transverse swelling ratio, *η*_T_ = *w*_*s*_/*w*_0_) relative to the isotropic case (see Fig. 4a), since the fibers do not provide significant reinforcement perpendicular to their alignment. The swelling anisotropy (defined as *η*_T_*/η*_L_) therefore increases with increasing fiber volume fraction (Fig. 4b). Accordingly, *w* will increase more than *L* upon exposure to a solvent, and the key geometric parameter *w/L* that defines the nonlinear behavior of the beam can be altered merely by exposing it to a suitable solvent. The swelling anisotropy *(η*_T_*/η*_L_) also determines the range in parameter space, Δ*w/L*, through which the geometry of a beam is able to move based on such swelling. The choice of the initial beam geometry *w*_0_*/L*_0_ at fabrication determines whether this range is sufficient to bring the beam’s geometry through the bifurcation (i.e., the *k*_1_ = 10 phase boundary in Fig. 3d for our 15 vol% PDMS-GF), as necessary to trigger an actuation event (Fig. 1c). As a proof of concept, we provide simple demonstrations of such autonomous actuation in Supplementary Movies 2 and 6 for material printed from PDMS-GF and exposed to toluene. In Supplementary Movie 2, a bistable unit actuates and jumps dramatically due to the rapid release of strain energy from the buckled beams. In Supplementary Movie 6, a bistable unit is integrated with the lid of a 3D printed box, and the strain energy is harnessed to open the lid when an appropriate solvent is encountered. In this case, the box remains closed when placed in water, but when the water becomes contaminated by the addition of toluene, the beams actuate and open the box lid. Using this approach, for example, a box could float indefinitely in the ocean and wait to autonomously open to release a chemical or to obtain a sample when a particular pollutant is encountered, without using batteries or sensors.

### Controlling the time of actuation

The distance between *w*_0_/*L*_0_ and the bifurcation *B* will set a time interval between the introduction of the stimulus (*t* = 0) and the time at which the beam actuates (*t* = t^*^). To quantify this time, we measured the changing value of *w/L* during solvent swelling (Fig. 4c) for the PDMS-GF15 ink during submersion in toluene. We normalize the beam’s changing *w/L* ratio (see also Supplementary Figure 6 for non-normalized data) by the initial ratio *w*_0_/*L*_0_ to provide a relative value, (*w*/*L*)_rel_ = (*w*/*L*)/(*w*_0_/*L*_0_), which, assuming simple diffusion, can be derived (Supplementary Note 7) as

1 $$\left( {w{\mathrm{/}}L} \right)_{{\mathrm{rel}}} = \left\{ {\begin{array}{*{20}{l}} {1 + \sqrt {\left( {t{\mathrm{/}}t_{\mathrm{s}}} \right)} \left( {\eta _{\mathrm{T}} - 1} \right)} & {,t < t_{\mathrm{s}}} \\ {\eta _{\mathrm{T}}} & {,t \ge t_{\mathrm{s}}} \end{array}} \right.$$

where the time *t*_*s*_ = *w*_0_^2^/8*D* is the time we expect the beam to be saturated (based on diffusion), and *D* is the diffusion coefficient of toluene in PDMS. Using equation (1) (Supplementary Note 7), the time at which we expect the beam geometry *w*/*L* to reach the bifurcation point *B* (and hence to actuate) is

2 $$\hat t^\ast = \frac{{\left( {BL_0 - w_0} \right)^2}}{{8D\left( {\eta _{\mathrm{T}} - 1} \right)^2}}$$

(note, we use $\hat t^ \ast$ to indicate the time of actuation predicted by equation (2), and t^*^ to indicate the measured time). Fig. 4d and Supplementary Movie 7 demonstrate how different *w*_0_/*L*_0_ values produce different t^*^ in accordance with equation (2). We have printed samples with widths of 600–850 μm and lengths of 7–9 mm. The actuation time for these can range from about 0.6 s to 108 s, which almost spans the range of theoretically predicted times (0–130 s) by equation (2).

Since *B* and *η*_T_ are unitless and *D* is an intrinsic parameter, the maximum actuation time for a beam with slenderness ratio *w*_0_/*L*_0_ would scale like *w*_0_^2^. For example, if the beams were made an order of magnitude smaller (e.g., around 85 μm in width) the range of available actuation times would decrease from roughly 0–130 s to 0–1.3 s. For any length scale, the theoretical upper bound of the actuation time is the time to saturation, *t*_*s*_ = *w*_0_^2^/8*D*, and the theoretical lower bound can be arbitrarily close to zero, as *w*_0_/*L*_0_ can always be chosen to satisfy *B-w*_0_/*L*_0_ →0. In reality, small perturbations in environmental conditions and fabrication limitations in beam geometry make infinitesimal $\hat t^ \ast$ unachievable and increase the uncertainty in experimental actuation time at these smaller values.

Despite the simplistic assumptions in the model, we found that the average discrepancy between $\hat t^ \ast$ and t^*^ was about 17% for samples designed to actuate more than 20 s after exposure to the stimulus. As this time is reduced, the relative error of the model increases. For example, for samples designed to actuate at times less than 5 s after exposure to the stimulus, the relative error of the model increases to about 44%. The high discrepancy for short actuation times (<5 s) arises from several challenging factors: non-uniform exposure to the chemical stimulus (not accounted for in the model, which assumes instantaneous, uniform exposure); the particularly close proximity of the geometric parameters to a bifurcation point when short delay times are desired, corresponding to a very small energy barrier; experimental uncertainty in the precise location of the phase boundary (exacerbated by the small energy barrier near bifurcation); and deviations in friction between the material and the substrate. At longer intervals of time (>5 s) these problems are diminished. In practice, this uncertainty would set a practical limit on how closely a sequence of ordered actuation events could be temporally spaced.

If we allow time for the solvent to evaporate, the beams return to their initial geometric parameters (*w*_0_/*L*_0_), and are once again bistable (see Supplementary Figure 7, and Supplementary Table 1). External energy is required to reset the units to the higher-energy state in order to reuse them. At ambient conditions, the drying time is about 70 min (though this could be smaller or larger depending on the length scale of the system) after which the units can be reset to the higher-energy state by compression (to buckle the beams again). We performed repeated tests (actuation–drying–resetting–actuation) on five units and found that the intra-sample variability of actuation time is comparable with the inter-sample variability, as discussed above (see Supplementary Table 2).

### Demonstrations of embodied logic

In addition to the control of actuation time, self-actuating systems that consist of multiple actuating units (each of which may be a different material, to respond to different stimuli) can be constructed. There are many ways that these can be arranged, leading to different possible system functions of varying complexity. We utilize a shorthand to describe some of these possible functions: first, we indicate a functional event in bold followed in parentheses by the condition that is necessary for that event to occur; example functions include **Open** and **Close**, referring, e.g., to the opening or closing of a box. Second, the application of a stimulus is indicated by the symbol S with an appropriate subscript; here, stimuli can include S_toluene_ (which actuates PDMS-based materials), S_water_ (which actuates hydrogel-based materials), and S_mass_ (which refers to placing a mass on the structure). For example, **Open**(S_water_) would indicate that a structure should open when exposed to water, which would be accomplished by using a hydrogel-based material to actuate. Finally, if multiple stimuli must occur within some time interval, this is indicated by a value assigned to Δt^*^. As a first example, we 3D print a box (see Methods) which is designed to autonomously open when exposed to toluene, but then to close again after a defined interval of time (e.g., 20 s). This behavior can be written as **TimedOpen**(S_toluene_ | Δt^*^ = 20 s). To open and then close the box, we need two actuation events, which we accomplish by integrating two PDMS-GF actuating units with the box, each with different values of *w*_0_/*L*_0_. These values are chosen such that simultaneous exposure to toluene will cause them to reach the phase boundary, *B*, 20 s apart. We show this behavior in Supplementary Movie 8: after exposure to toluene, the right unit actuates first, opening the box. Then, after ~20 s, the left unit actuates and closes the box. The second demonstration mimics the rapid closing of the Venus flytrap when an object (denoted S_mass_) is placed on a waiting ledge, with the added requirement that the trap is only in operation if a chemical signal (toluene) has been applied (see Supplementary Movie 9). We can represent this behavior as **Close**(S_toluene_ ∧ S_mass_). The trap is locked until a PDMS-based unit actuates, preventing the lobes from closing until *t* ≥ t^*^ even if a load is applied before then.

**Fig. 5:**
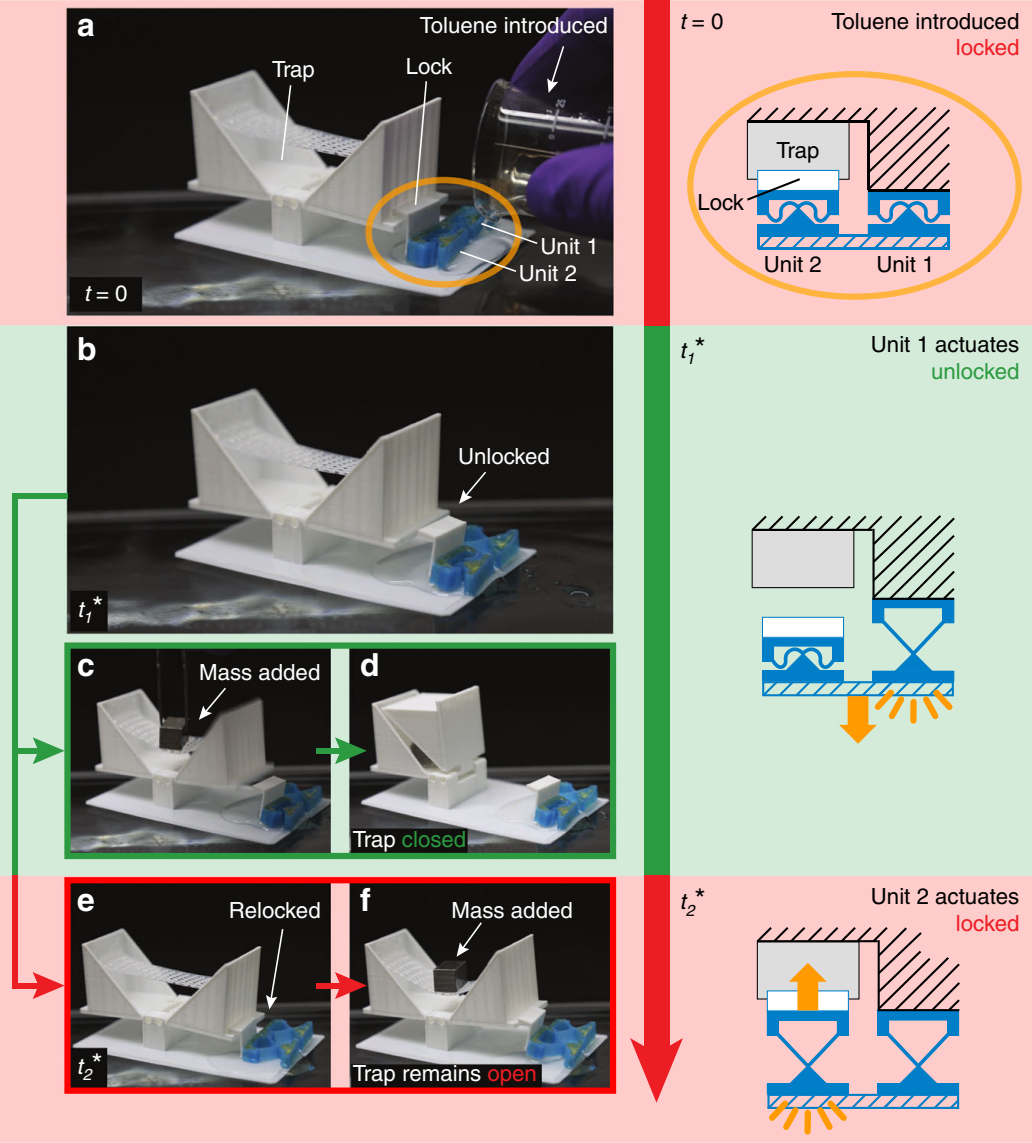
A “flytrap” with embodied logic **Close**(S_toluene_, S_mass_ | $t_{{\mathrm{mass}}}^ \ast$ - $t_1^ \ast$ < $t_2^ \ast$ - $t_1^ \ast$). **a** Toluene is applied to a flytrap-inspired system which is prevented from closing by a lock that is toggled by two actuating units. **b** The unit with larger *w*_0_/*L*_0_ actuates first (at time $t_1^ \ast$), which unlocks the lobe. **c**, **d** If a mechanical load is applied while the flytrap is unlocked it closes. **e**, **f** Later, the second bistable unit actuates (at time $t_2^ \ast$) and relocks the lobe; adding a mechanical load at this point has no effect

Figure 5 shows a similar Venus flytrap-inspired example, but with more complex control logic. In this example, there are two actuating units made from PDMS-GF, which are designed to actuate 10 s apart (Fig. 5a). The first of these (which actuates at $t_1^ \ast$) activates the system by removing the lock (as in Fig. 5b) while the second (which actuates at $t_2^ \ast$) re-locks the flytrap, once again preventing it from closing. If a mass is placed on a platform in the center of the flytrap at a time between $t_1^ \ast$ and $t_2^ \ast$ (i.e., when the lock is disengaged) the lobes will close (Fig. 5c, d and Supplementary Movie 10). If the mass is placed on the platform when the lock is engaged (e.g., after $t_2^ \ast$, as in Fig. 5e, f and Supplementary Movie 11) it will have no effect. This functionality is described by **Close**(S_toluene_, S_mass_ | $t_{{\mathrm{mass}}}$ - $t_1^ \ast$< $t_2^ \ast$ – $t_1^ \ast$), where the comma indicates an ordered list of stimuli, and $t_2^ \ast$–$t_1^ \ast$ = 10 s.

**Fig. 6:**
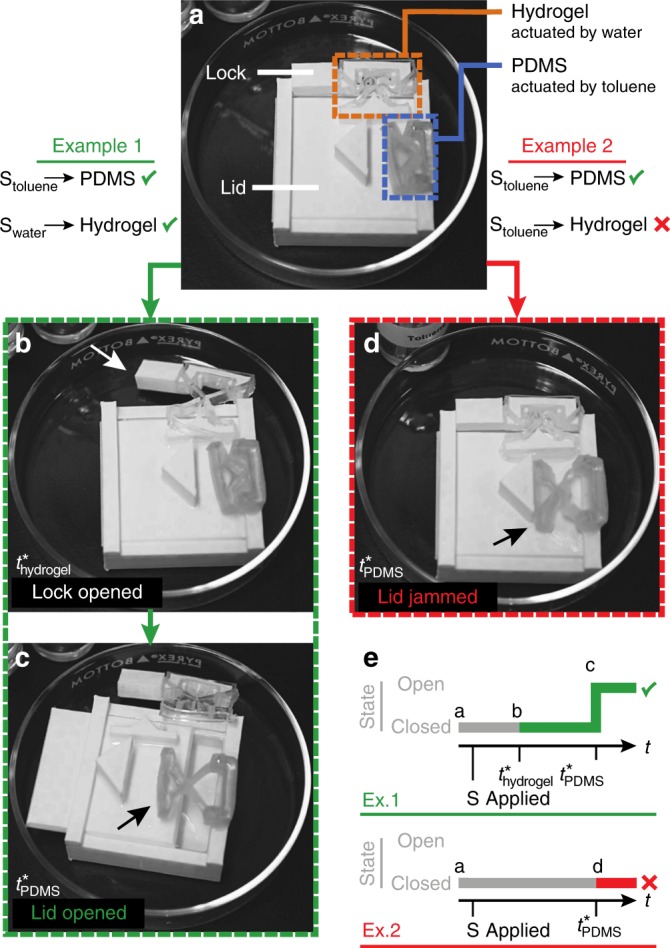
Multimaterial responsive systems, e.g., **Open**(S_water_ ∧ S_toluene_ | $t_{{\mathrm{hydrogel}}}^ \ast$ < $t_{{\mathrm{PDMS}}}^ \ast$). **a** A box with a lid which is unlocked upon actuation of a hydrogel-based unit (at time $t_{{\mathrm{hydrogel}}}^ \ast$) and subsequently opened upon actuation of a PDMS-based unit (at time $t_{{\mathrm{PDMS}}}^ \ast$). **b**, **c** Example 1: if water is applied to the hydrogel-based unit and toluene to the PDMS-based unit, the lid is unlocked and then pushed open, successfully opening the box (Supplementary Movie 12). **d** Example 2: if toluene is applied to both units, the PDMS-based unit actuates as it should, but because the hydrogel-based unit is unresponsive to toluene, the lock interferes and the lid remains closed (Supplementary Movie 13). **e** Diagram representing the system logic and the behaviors of the system in Examples 1 and 2

Finally in Fig. 6, we demonstrate a multi-stimuli-responsive system^67,68^, which uses two materials that respond to two orthogonal stimuli (i.e., hydrogel, which responds to water, and PDMS, which responds to a non-polar solvent such as toluene). We compose a box which follows the behavior **Open**(S_water_ ∧ S_toluene_ | $t_{{\mathrm{hydrogel}}}^ \ast$ <$t_{{\mathrm{PDMS}}}^ \ast$), as shown in Fig. 6a. Application of S_water_ causes the hydrogel-based unit to actuate, opening a lock (at time $t_{{\mathrm{hydrogel}}}^ \ast$) that otherwise obstructs the opening of the lid. Application of S_toluene_ causes the PDMS-based unit to actuate (at time $t_{{\mathrm{PDMS}}}^ \ast$), pushing open the lid if it has been unlocked (Fig. 6b, c and Supplementary Movie 12). If instead we apply only toluene, the hydrogel-based lock does not respond, and the box remains closed even when the PDMS-based unit pushes on the lid (Fig. 6d, e and Supplementary Movie 13). This capability could be used, for example, to produce a sampling box that autonomously opens when it encounters an oil–water interface, without any external power or solid-state sensors and actuators.

These principles can be further extended to 2D or 3D by designing more complicated arrangements of beams. As a demonstration of this, we manufactured 2D samples using PDMS-based materials and illustrated autonomous deployment due to the presence of toluene (Supplementary Movie 14).

## Discussion

In summary, we have designed and 3D printed systems of self-actuating structures capable of simple logic (AND, OR, and NAND) and controlled timing of actuation in response to multiple stimuli. We have accomplished this using PDMS-based and hydrogel-based materials that respond to different environmental stimuli. Due to the use of short fiber composites and the alignment of the fibers during extrusion, the beams swell anisotropically in the presence of suitable stimuli, triggering rapid and large-amplitude configuration changes at predetermined times (as the geometric parameter *w*_0_/*L*_0_ passes a bifurcation point). Using solely architected soft materials as self-actuating functional elements, our approach enables complex function and control of timing in response to multiple stimuli. We demonstrated several autonomous responsive systems in this work as examples of our approach, all performing their designated functions without mechatronics, traditional control systems, or tethering. This includes a hopper that autonomously jumps when an undesired chemical is introduced, a “flytrap” that only closes if mechanically stimulated during a pre-programmed interval of time, and a box that only opens if it encounters both non-polar solvents and water. While this work focuses on the autonomous release of strain energy to produce precise actuation events, these concepts could be integrated with more complex systems. For example, advances from soft robotics, such as the integration of pneumatic or chemical energy sources^69^, could allow the units to be autonomously reset to allow repeated actuation events. We also note that the nonlinear behavior of the beams (e.g., the location of the bifurcation points in parameter space) is scale-independent, and therefore the systems have the potential to be scaled down or up as may be necessary for additional applications, e.g., in soft robotics, biomedical devices, and deployable structures. Finally, we made use of materials that respond to non-polar solvents or to water, but the same concept would apply to matrices that respond to other cues (e.g., light, temperature, and electric potential) as long as the printed materials are anisotropic.

## Methods

### Ink preparation and 3D printing

PDMS and glass fibers are mixed under vacuum, and then transferred to a syringe and centrifuged (see more discussion in Supplementary Information). We use nozzles with an inner diameter of 410-μm for PDMS-based ink printing in this work. The hydrogel has an N-isopropylacrylamide (NIPAm) network and nanofibrillated cellulose (NFC) as a filler. After preparation of the ink (discussion in Supplementary Information), it is printed through a nozzle of diameter 250-μm. To improve the bistable response, two parallel filaments of the hydrogel-based ink are printed and then PDMS is injected in between to form a hydrogel beam. A 3D translation stage controls motion of the nozzle during printing. The PDMS-based ink is thermally cured and epoxy is cast and cured to provide desired boundary conditions. The hydrogel-based ink is cured by UV cross-linking and then mounted on 3D printed polylactic acid (PLA) pieces. We used fused deposition modeling (FDM) for fabrication of parts for demonstrations, with the functional PDMS or hydrogel units integrated with these by hand. The parts are fabricated on a MakerGear M2. We use a nozzle of diameter 0.25 mm at an extrusion temperature of 190 °C, a bed temperature of 65 °C, and speeds of 30–80 mm/s.

### Mechanical testing

We performed tensile tests on an Instron Model 5564 (displacement control), with tensile bars printed in either longitudinal or transverse directions relative to the loading direction. The nominal strain rate is constant at 1% for all tests. The actuation time associated with the snap-through of the structures was measured using a high-speed camera (Photron AX200) at 2000 frames per second. We performed continuous shear-rate ramp and stress sweep rheology tests on a rheometer (TA Instruments^®^ AR2000) at ambient temperature using a 20 -mm parallel plate with a 140-μm gap size for both PDMS-GF and hydrogel-based inks.

## Supplementary information

Supplementary Information

Description of Additional Supplementary Files

Supplementary Movie 1

Supplementary Movie 2

Supplementary Movie 3

Supplementary Movie 4

Supplementary Movie 5

Supplementary Movie 6

Supplementary Movie 7

Supplementary Movie 8

Supplementary Movie 9

Supplementary Movie 10

Supplementary Movie 11

Supplementary Movie 12

Supplementary Movie 13

Supplementary Movie 14

## Data Availability

All data generated or analyses during this study are included in this published article (and its supplementary information files).
