# Supplementary material for: Bifurcation-based embodied logic and autonomous actuation
Source: Nat Commun. 2019 Jan 10;10:128. doi: 10.1038/s41467-018-08055-3 (PMC6328580; doi:10.1038/s41467-018-08055-3)
Supplement: Supplementary file 1 — Supplementary Information [file 41467_2018_8055_MOESM1_ESM.pdf]

Supplementary Information

Bifurcation-based embodied logic and autonomous actuation

Yijie Jiang, Lucia M. Korpas, and Jordan R. Raney*

Department of Mechanical Engineering and Applied Mechanics, 220 S 33rd St., University of Pennsylvania, Philadelphia, PA 19104, USA

Corresponding author: Jordan R. Raney raney@seas.upenn.edu

Supplementary Figures

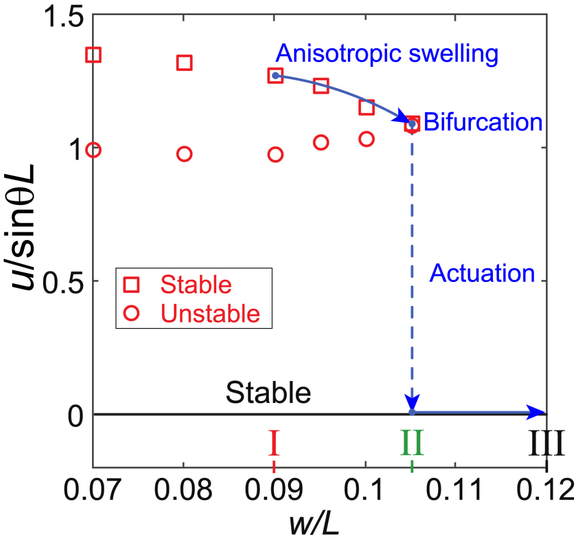

Supplementary Figure 1 | Bifurcation diagram for the geometric parameter *w/L*. The diagram is derived from finite element simulations and indicates the normalized displacements at which critical points (local energy minima and maximum) exist. The arrow illustrates how anisotropic swelling could lead to actuation due to bifurcation. The positions of Roman numerals (I-III) correspond to those in Fig. 1c.

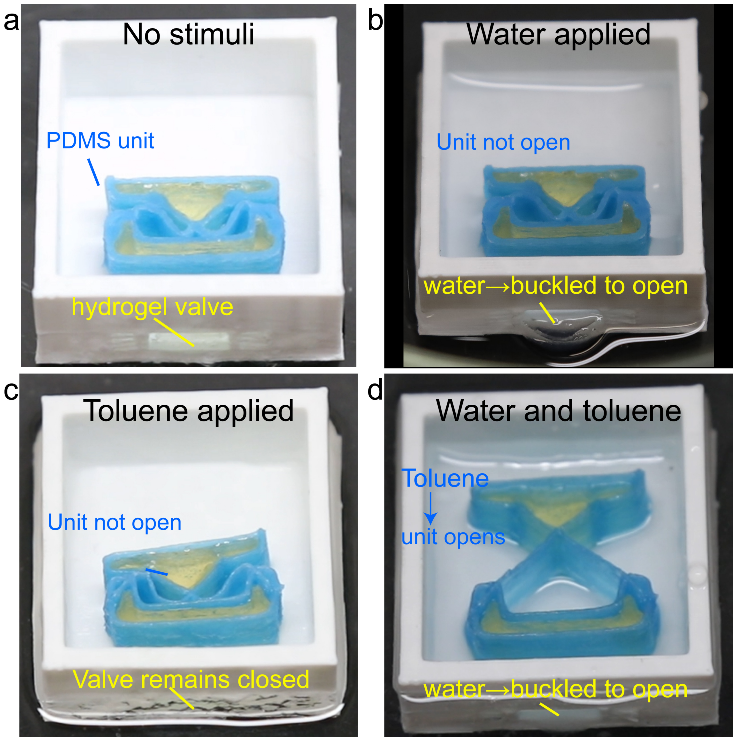

Supplementary Figure 2 | Demonstration of AND gate behavior. The box has a PDMS unit inside and a hydrogel valve, which is closed. When exposed to water, the hydrogel swells and buckles to open the valve, allowing solvent to go into the box. **(a)** No stimuli are present. **(b)** When there is only water, the valve opens and the water reaches the inner unit, but it cannot actuate the unit. **(c)** When only toluene is applied, the hydrogel valve remains closed and the toluene cannot reach the inner unit. **(d)** When water and toluene are both applied, the valve opens, toluene and water go into the box, and the toluene is absorbed into the unit, causing actuation to occur.

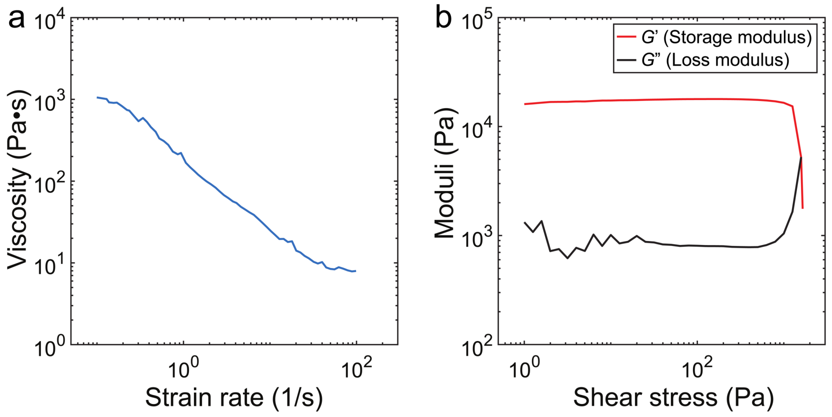

Supplementary Figure 3 | The rheological properties of hydrogel-cellulose fibril ink. It possesses a rheology that **(a)** is shear-thinning (exhibits a decrease in the apparent viscosity with increasing strain rate) and **(b)** includes a viscoelastic yield stress behavior, with a pronounced drop in the storage modulus at high shear stress.

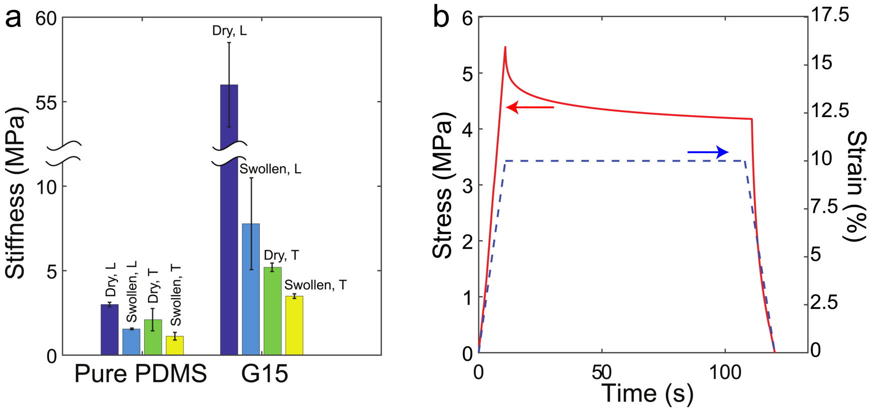

Supplementary Figure 4 | Additional mechanical measurements on PDMS-GF15 material. (a) Results of tensile tests on dry and solvent-swollen tensile bars printed in longitudinal and transverse directions for both pure PDMS and PDMS-GF15 inks. **(b)** Stress relaxation tests for the PDMS-GF15 composite.

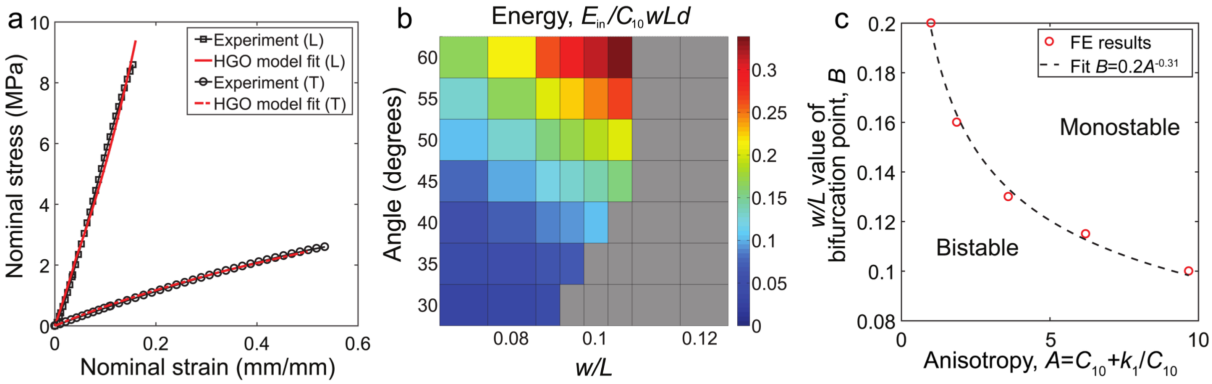

Supplementary Figure 5 | Finite element mechanical model and bistability simulations. (a) Nominal tensile stress-strain curves for PDMS-GF15 materials printed longitudinally (fibers parallel with loading) and transversely (fibers perpendicular to loading) and fitted using the HGO model. **(b)** Strain energy of the elastic beam at the peak of the energy barrier between the two stable states. The gray area indicates a monostable beam (only stable at u=0). **(c)** The location of the bifurcation point, B, as a function of material anisotropy as predicted by the HGO model (for a beam tilted at 45º), empirically following power law behavior.

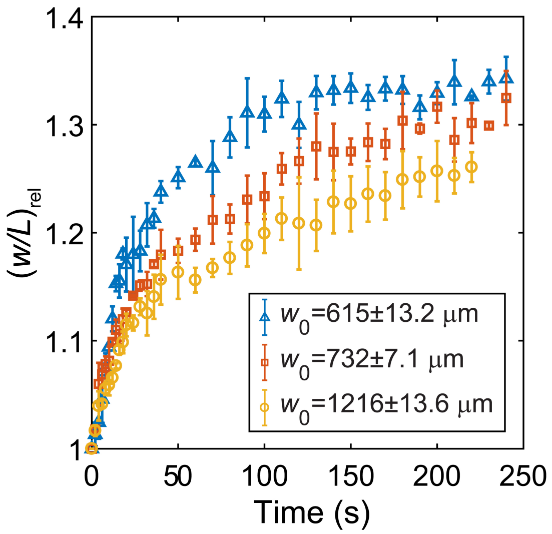

Supplementary Figure 6 | The relative change in *w*/*L* for PDMS-GF15 beams as a function of time. The measurements are for three beams with different initial widths. The error bars are the standard deviation of three measurements.

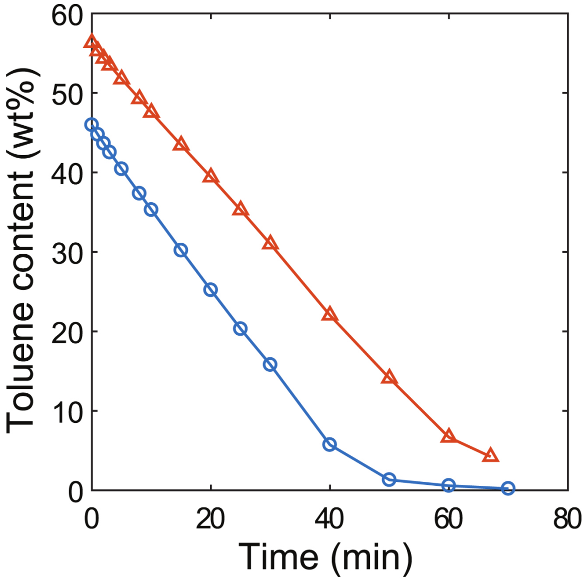

Supplementary Figure 7 | Evaporation of toluene from PDMS-GF15 beams. Measurement of the weight percentage of toluene in soaked samples evaporating in ambient as a function of time.

Supplementary Tables

Supplementary Table 1 Measurements of *w* for two PDMS-GF15 beams before submerging in toluene and after toluene is fully evaporated.

Beam #	Beam width (μm)	
	Before Toluene	After Toluene evaporation
1	775.4 ± 28.95	725.4 ± 43.63
2	759 ± 39.24	764.3 ± 28.06

Supplementary Table 2 Repeatability of actuation of PDMS-GF15 units. At least 70 min (ambient) was allowed between each test to ensure evaporation of all toluene.

Sample #	Repetitions	Actuation time (s)
1	11	34.42 ± 6.13
2	11	16.34 ± 3.74
3	11	2.27 ± 0.92
4	6	11.24 ± 3.97
5	6	3.05 ± 0.54

Supplementary Notes

Supplementary Note 1: DIW 3D printing

Direct ink writing (DIW) is an extrusion-based 3D printing technique [1,2] in which material is extruded from a translating deposition nozzle at ambient conditions, relying on non-Newtonian material rheology to produce a pattern that maintains its shape after extrusion (Supplementary Figure 3). By decoupling the patterning step from the material cross-linking step, it offers a materials flexibility that allows us to meet all four criteria listed in the main text. For this approach to work it is important that the material is shear-thinning (to facilitate easy extrusion from a fine nozzle), and that it possesses a viscoelastic yield stress (so that the material maintains its shape after it is deposited). Subsequent immobilization steps (thermal or UV crosslinking, sintering, etc.) can then be taken after the pattern is formed, as appropriate for the specific material, to obtain the desired materials properties.

Supplementary Note 2: Additional mechanical measurements on PDMS-based composites

Tensile bars using pure PDMS and PDMS-GF15 inks were fabricated by direct ink writing. Both longitudinal and transverse samples were prepared (i.e., fibers oriented parallel with and perpendicular to the loading direction, respectively). After curing, some samples (referred to as “swollen” in Supplementary Figure 4a) were soaked in solvent (toluene) for 24 h, and other samples were not exposed to solvent (“dry” in Supplementary Figure 4a). Subsequently, an Instron Model 5564 was used to perform tensile tests on these samples in displacement control at a nominal strain rate 0.01 s^{-1} for all samples. Three samples were tested for each case (Supplementary Figure 4a). The swollen samples, infused with solvent, showed lower stiffness compared with the dry samples, but in both cases the materials remained highly anisotropic (i.e., for the G15 case in Supplementary Figure 4a notice the decrease in stiffness going from “Dry, L” to “Dry, T” but also going from “Swollen, L” to “Swollen, T”). We also conducted stress relaxation tests (Supplementary Figure 4b) to confirm that the material was sufficiently elastic to maintain the modest strains associated with buckling without prohibitive viscoelastic relaxation. An Instron MicroTester Model 5848 was used, with a ramp to 10% strain, followed by a hold for 100 s and

subsequent unloading. Stress relaxation was observed during the hold (Supplementary Figure 4b), such that the stress, σ, could be approximated by a power-law function, $\sigma = Kt^m$. For pure PDMS and PDMS-GF15 inks, the fitted exponents are m_{PDMS}=-0.0095±0.0016 and m_{GF15}=-0.023±0.008, respectively. Stress relaxation tests were performed at material strains comparable to those experienced during buckling, and these indicate a stable elastic response, ensuring that the beams can store elastic strain energy in a buckled configuration without prohibitive relaxation, as they must prior to actuation.

Supplementary Note 3: Halpin-Tsai model for short fiber composites

The Halpin-Tsai model[3,4] is a micromechanics model commonly used for composites that include short, aligned fibers. It provides expressions for the stiffness along two principal directions relative to the fiber direction: longitudinal (L, parallel with fibers) and transverse (T, perpendicular to the fibers):

$$E_i = E_{\mathrm{m}} \frac{1+\beta_i \xi_i f}{1-\beta_i f} \tag{1}$$

where f is the volume fraction of fibers, E_{f} and E_{m} are the Young's modulus of the fibers and the matrix, respectively, i indicates the direction (either L or T), and β_i is defined as

$$\beta_i = \frac{E_{\mathrm{f}}/E_{\mathrm{m}}-1}{E_{\mathrm{f}}/E_{\mathrm{m}}+\xi_i} \tag{2}$$

L_{f} and d_{f} are the length and diameter of the fibers, respectively, and we use ξ_{L}=10 in our fitting. For the PDMS-based composite with 15 vol% glass fibers (PDMS-GF15), when the values for E_{f} and E_{m} are obtained from fitting the longitudinal and transverse experimental data to Supplementary Equation (1) and (2), the results are E_{f}=52.13 GPa and E_{m}=2.96 MPa. Both values are consistent with glass and PDMS material properties.

Supplementary Note 4: Experimentally-determined phase boundary

To determine the phase boundary experimentally, we fabricated samples with a fixed angle of 45° and systematically-varying w/L ratios. Each sample was manually compressed to the buckled state and then released. Bistable samples remained in their buckled configuration, while monostable samples snapped

back to their undeformed configuration upon release of the load. We found that the maximum *w/L* for bistable units (with beam angle 45°) was 0.102 and the minimum *w/L* for monostable units was 0.108. Thus, we determined the phase boundary is 0.102 < *w/L* < 0.108 (with fabrication inconsistencies causing the range).

Supplementary Note 5: Finite element analysis (FEA) on anisotropic bistable structures

Prior work[5] determined the relationship between beam geometries (θ and *w/L*) and the stability behavior (bistable or monostable) for isotropic material. However, in addition to geometric parameters, the degree of material anisotropy also influences the stability. As a result, the geometric boundaries between behavioral regimes (e.g., bistable vs. monostable) that were determined previously are not universal and do not apply to anisotropic materials. Therefore, we used FEA to locate the boundary between regions of monostability and bistability in the geometric phase diagram (Fig. 1d) for the more general case in which the material anisotropy is allowed to vary. Before simulating the beams, we first fit an anisotropic hyperelastic mechanical model, the Holzapfel-Gasser-Ogden (HGO) model[6] , which expresses the strain energy as

$$U = C_{10}(\overline{I_1} - 3) + \frac{1}{D}\left(\frac{(J^{\mathrm{el}})^2}{2} - \ln J^{\mathrm{el}}\right) + \frac{k_1}{2k_2}\sum_{\alpha=1}^{N}\{\exp[k_2\langle\overline{E_\alpha}\rangle^2] - 1\} \tag{3}$$

with

$$\overline{E_\alpha} \stackrel{\mathrm{def}}{=} \kappa(\overline{I_1} - 3) + (1 - 3\kappa)\left(\overline{I_{4(\alpha\alpha)}} - 1\right) \tag{4}$$

where U is the strain energy per unit volume, C_{10}, D, k_1, k_2, and κ are material parameters that determine the mechanical behavior and need to be provided by the user. More details about this model and the detailed definitions of the other parameters can be found in the Abaqus manual.

To determine the parameters for the PDMS-GF15 material, tensile tests in both the longitudinal and transverse directions were simulated using a range of HGO material parameters, generating stress-strain curves that were fit to the experimental stress-strain data (Supplementary Figure 5a). The best-fit

parameters were C_{10}=1.1525 MPa, D=7.805×10^{-3} MPa^{-1}, k_1=10 MPa, k_2=0.01, and κ=0 (see Supplementary Figure 5a). The anisotropy of the material can be indicated by $(C_{10}+k_1)/C_{10}$ with larger values of k_1 corresponding to increasing anisotropy. If k_1=0, the material is isotropic.

Beams with different *w/L* and tilt angle θ were modeled in Abaqus/Explicit® (version 6.9) using 2D plane strain models with CPE4R elements. The beams had fixed length 10 mm, systematically varied *w/L* from 0.07 to 0.12, and θ from 30° to 60°. Each tilted beam had one fixed end and was deformed by applying a vertical displacement to the other end, while constraining the horizontal displacement. The results of simulations of a tilted beam (θ=45°) with *w/L* varying from 0.07 to 0.12 are shown in Fig. 3a and 3b. The strain energy is normalized as $E/(C_{10}wLd)$, the force is normalized as $F/(C_{10}\cos\theta Ld)$, and the displacement is normalized as $u/(L\sin\theta)$, where C_{10} is a fitting parameter in the HGO model and d is the out-of-plane thickness of the beam, which is set to unity. A beam is bistable if its strain energy, simulated as a function of displacement, is found to have a second energy minimum for some nonzero value of u. For the value of C_{10} corresponding to the 15 vol% PDMS-GF material, and with a beam angle of θ=45°, the FEA shows that the beam is bistable if *w/L* is less than 0.105, and monostable otherwise. This FEA result is consistent with our observations with the 3D printed beam structures, for which we have experimentally determined a phase boundary between $0.102<w/L<0.108$ for θ=45°. We can develop a more complete phase diagram using FEA by systematically varying θ and *w/L*. We plot the FEA results of the strain energy of the elastic beam at the peak of the energy barrier, relative to the two stable morphologies (E_{in} indicates the strain energy of the peak relative to the unstrained beam at u=0 and E_{out} indicates the peak energy relative to the second, higher-energy stable state, as shown schematically in Fig. 1b). These quantities are normalized as $E_{in}/(C_{10}wLd)$ in Supplementary Figure 5b and $E_{out}/(C_{10}wLd)$ in Fig. 3c. The grey areas indicate regions of monostability while the different colors indicate bistability (i.e., E_{out} exists and is greater than 0). We performed a parametric study to determine the effect of the degree of anisotropy on the location of the bifurcation, B for different θ and *w*/*L* (forming phase boundaries). With increasing material anisotropy, the *w/L* value of the boundary at a given angle decreases as a power law function (see Supplementary

Figure 5c for the case θ=45°). For a beam of fixed length, L, the greater the degree of material anisotropy, the thinner the beam must be to be bistable. Figure 3d shows the bistable/monostable phase boundary for different degrees of material anisotropy: the isotropic case (k_1=0) is indicated by the black line, with increasing degrees of material anisotropy corresponding to larger values of k_1. When we set k_1=0 (i.e., isotropic) in the HGO model, we recover the boundary expected for a Neo-Hookean isotropic material, as used in the previous study [5]. For the anisotropic material used here (e.g., the PDMS-GF15 ink), the boundary for a beam with a 45° tilt angle moves from w/L = 0.2 for an isotropic material to w/L = 0.105, i.e., nearly a factor of two.

Supplementary Note 6: Beam swelling measurements

Once the material is fully saturated by the solvent, the swelling is at a maximum such that, for an isotropic material, every linear dimension will have increased by the same factor. This factor is the *swelling ratio*, $\eta = x_s/x_0$ (where x_0 indicates the initial length of the material along some linear dimension and x_s indicates the length along that same dimension after the material is completely saturated with solvent). The value of the swelling ratio is unique to each material-solvent combination, based on the chemical affinity.

Three PDMS-GF15 beams with different initial widths were fabricated and submerged in toluene. The values of w and L were recorded as a function of time by recording video using an optical microscope (Amscope® MU1000) and post-processing using ImageJ. Time t=0 is defined as the first stable frame after the toluene was added. As shown in Supplementary Figure 6, $(w/L)_{rel} = (w/L)/(w_0/L_0)$ increases quickly initially and then converges to a constant value at longer times. Since the diffusion time depends on the amount of material through which the solvent must diffuse, the initial beam width affects when the equilibrium value is reached. To determine swelling at saturation, microscope images were taken before and after one day of submersion in toluene, allowing assessment of initial and final geometries using ImageJ.

Supplementary Note 7: Beam swelling model and estimated actuation time

When there is enough solvent for a material to absorb, the material eventually becomes fully saturated with the solvent, defining the saturation time, t_s (which depends on the beam volume through which the solvent diffuses). For $t \geq t_s$, the quantity $(w/L)_{rel}$ therefore does not change, remaining constant at a value corresponding approximately to the swelling ratio for the specific material-stimulus combination, η_T/η_L. For the time prior to this ($t<t_s$), we model the observed changes to $(w/L)_{rel}$ by assuming a mechanism of simple diffusion. The beams are printed with significant out-of-plane thickness, d (see photos of physical samples in Figs. 5 and 6). The solvent front is therefore assumed to diffuse inward from the two surfaces in the L-d plane, perpendicular to the long axis, L, and parallel with the direction of the beam width. Assuming simple diffusion, the location of the solvent front relative to these surfaces increases in depth, a, as

$$a = \sqrt{2Dt} \tag{5}$$

where D is the diffusion coefficient (e.g., of toluene in PDMS). The time at which the beam is entirely saturated, t_s, occurs when the two solvent fronts diffusing from the two surfaces meet in the middle of the beam, i.e., when $a=w_0/2$, which occurs at time

$$t_s = \frac{w_0^2}{8D} \tag{6}$$

We assume that as the front propagates into a new infinitesimal slice of the beam, dw, that portion of the beam immediately takes on the saturated dimensions $dw=\eta_T dw_0$. The length remains approximately L_0 since the swelling ratio in the longitudinal direction is very close to $\eta_L=1$ for our PDMS-GF15 material (Fig. 4a). Then the changing value of $(w/L)_{rel}=(w/L)/(w_0/L_0)$ follows the relationship

$$(w/L)_{\text{rel}} = \begin{cases} 1 + \sqrt{(t/t_{\text{s}})}(\eta_{\text{T}} - 1), & t < t_{\text{s}} \\ \eta_{\text{T}} & , t \geq t_{\text{s}} \end{cases} \tag{7}$$

where η_T is the swelling ratios in the transverse direction (as found experimentally and shown in Fig. 4a). By fitting our experimental measurements to this model as shown in Fig. 4c for PDMS-GF15 (see also the experimental data in Supplementary Figure 6), we find D=(6.93±0.5)×10^{-6} cm^{2}s^{-1}, which is close to the

value reported for toluene in a different type of PDMS[7,8]. We use this to derive a time, $\hat{t}^*$, at which the model predicts that the beam geometry will reach the bifurcation point (i.e., the expected time of actuation). By noting that $w/L=B$ at $t=\hat{t}^*$ and expressing w/L as $(w_0/L_0)(w/L)_{\mathrm{rel}}$, we combine Supplementary Equation (6) and (7) to find

$$\hat{t}^* = \frac{(BL_0 - w_0)^2}{8D(\eta_{\mathrm{T}} - 1)^2} \tag{8}$$

Supplementary Note 8: Recovery and reusability of PDMS-based beams

Based on the robust cross-linking of PDMS, we expected that after exposure to solvent and subsequent drying that all solvent would leave the material and allow the beams to be reused. To confirm this, three sections of different PDMS-GF15 beams were first weighed, and dimensions were recorded. They were then soaked in toluene for ~1 day. Then the materials were removed from the toluene and placed on a digital scale to allow measurement of the mass as a function of time. Supplementary Figure 7 shows the gradual reduction of toluene content inside the beams (calculated based on the ratio of mass of toluene, taking the difference between the instantaneous measured mass and the original dry mass, to the instantaneous mass). It takes ~70 min for all toluene to evaporate. Supplementary Table 1 shows measurements of the width of two PDMS-GF15 beams before submerging into toluene and over about 70 min of evaporation time, showing complete recovery of the original geometry within the margin of error. After the toluene evaporates, the beams move from the swollen, monostable configuration back to their original bistable geometries. With these structures once again exhibiting bistability, they can be reused for autonomous actuation in experiments.

Supplementary Note 9: Ink preparation

PDMS-based inks

PDMS (SE 1700 and Sylgard® 184, Dow Corning Co.) and glass fibers (Fibre Glast Co.) are mixed (SpeedMixer™, FlackTek, Inc) at 1500 rpm for 45 s, 1800 rpm for 30 s and 1200 rpm for 2 min under 20 Torr vacuum. The ratio of SE 1700 to Sylgard® 184 is 85:15 for 0 and 5 vol% glass fiber ink, and 83:17

for 10, 12, and 15 vol% glass fiber inks. The base to cross linker ratio is 10:1 for both SE 1700 and Sylgard® 184. The glass fibers are observed to be ~540 μm in length and 14.6±1.53 μm in width as received from the manufacturer, but the length decreases to 72.9±17.3 μm during mixing. The mixed ink is then transferred to a syringe and centrifuged (ST 8 Centrifuge, Thermo Scientific®) at 3400 rpm for 13 min, and a deposition nozzle is connected to the syringe barrel via Luer lock (Nordson EFD®). In this study, nozzles with an inner diameter of 410 μm are used for all PDMS-based ink printing.

Hydrogel-based inks

First, nanofibrillated cellulose (NFC) is diluted in DI water, which is deoxygenated under nitrogen flow for 30 min, and mixed at 800 rpm for 15 s and 1500 rpm for 1 min in a capped container. Synthetic hectorite clay (Laponite XLG) is then added under nitrogen flow and mixed at 1800 rpm for 3 min and 1500 rpm for 2 min. The clay solution is additionally hand mixed if white aggregates are seen. N-isopropylacrylamide (NIPAm) is then added to the clay solution under nitrogen flow and mixed again at 1800 rpm for 3 min and 1500 rpm for 2 min. Irgacure 2959, the ultraviolet photoinitiator, is added and the ink is mixed at 1500 rpm for 2 min. The mass percentage of these components are DI water: NFC: Laponite clay: NIPAm: Irgacure= 81: 0.77: 10: 8.1: 0.12. Finally, 5 wt% PDMS (Sylgard® 184 with 10% crosslinker) is added and mixed at 800 rpm for 1 min. The mixed ink is then transferred to a syringe and centrifuged at 3400 rpm for 13 min. A metal straight nozzle with outlet diameter of 250 μm is used for hydrogel-based ink printing. Because the hydrogel has a more time-dependent mechanical response than the printed PDMS, it was more challenging to obtain the desired bistable response. To improve this, each hydrogel beam was actually fabricated as a composite: first, two parallel filaments of the hydrogel-based ink were printed, separated by a small amount (on the order of the filament width), then PDMS (Sylgard® 184) was extruded in between. The PDMS provided a more robust elastic response of the overall beam, but the hydrogel still fully enclosed the PDMS so that the composite beam only becomes swollen in the presence of water.

Supplementary Note 10: DIW printing

A ShopBot® D2418 3D translation stage is used for control of movement of the syringe in 3D. Print paths are controlled by G-code or ShopBot® (G-code derivative) commands, which are generated by Python or Matlab® scripts. A volumetric dispenser (Ultra™ 2800, Nordson) or a pressure control box (EFD® Ultimus™ V) is used for control of ink extrusion flow rate. Samples are printed on a substrate of Teflon-coated aluminum foils at ambient temperature. PDMS-based samples are cured in an oven (HERATherm, Thermo Scientific®) at 100 ºC for 1 h. Hydrogel-based samples are cured via UV crosslinking (~60 mW/cm^2 for 300 s in OmniCure® Series 2000). Systems that include both materials are first UV cured and then thermally cured at 60 ºC for 4 h in a capped container. In order to provide the desired boundary conditions for the beams, epoxy (Epon 828, resin:cross linker=3:1) was cast and cured at room temperature for more than 6 hours to provide rigid boundaries for both ends of each PDMS beam. The hydrogel-based units were mounted on 3D printed polylactic acid (PLA) pieces, to provide appropriate boundary conditions. After completing these steps, we found that approximately 80% of the specimens possessed the desired mechanical functionality, while the other 20% did not. This yield rate is significantly lower than our previous work using isotropic materials. This therefore seems to be a result of the use of fibers (e.g., the fibers can partially clog the nozzle, producing clumping of the fibers in the material; the presence of fibers also roughens the surface of the beams; these inhomogeneities affect the mechanical properties; the higher the volume fraction, the more of an issue this becomes).

Supplementary References

1. Raney, J. R. & Lewis, J. A. Printing mesoscale architectures. *MRS Bull.* **40,** 943–950 (2015).
2. Lewis, J. A. Direct ink writing of 3D functional materials. *Adv. Funct. Mater.* **16,** 2193–2204 (2006).
3. Halpin, J. C. Effects of Environmental Factors on Composite Materials. *Tech. Rep. Afml-Tr-67-423* (1969).
4. Halpin, J. C. & Kardos, J. L. The Halpin-Tsai equations: A review. *Polym. Eng. Sci.* **16,** 344–352 (1976).
5. Shan, S. *et al.* Multistable Architected Materials for Trapping Elastic Strain Energy. *Adv. Mater.* **27,** 4296–4301 (2015).
6. Holzapfel, G. A., Gasser, T. C. & Ogden, R. W. A new constitutive framework for arterial wall mechanics and a comparative study of material models. *J. Elast.* **61,** 1–48 (2000).
7. Chao, K. P., Wang, V. S., Yang, H. W. & Wang, C. I. Estimation of effective diffusion coefficients for benzene and toluene in PDMS for direct solid phase microextraction. *Polym. Test.* **30,** 501–508 (2011).
8. Alexander, M., Boscaini, E., Lindinger, W. & Märk, T. D. Membrane introduction proton-transfer reaction mass spectrometry. *Int. J. Mass Spectrom.* **223–224,** 763–770 (2003).
