# Supplementary material for: Bifurcation-based embodied logic and autonomous actuation
Source: Nat Commun. 2019 Jan 10;10:128. doi: 10.1038/s41467-018-08055-3 (PMC6328580; doi:10.1038/s41467-018-08055-3)
Supplement: Supplementary file 2 — Description of Additional Supplementary Files [file 41467_2018_8055_MOESM2_ESM.pdf]

Description of Additional Supplementary Files

File Name: Supplementary Movie 1

Description: High-speed camera recording (2000 fps) of a snap-through event for a PDMS-based bistable unit after exposure to toluene (the structure is glued to the surface).

File Name: Supplementary Movie 2

Description: High-speed camera recording (2000 fps) of actuation and jumping of a bistable unit as the strain energy is released (the structure is not glued to the surface).

File Name: Supplementary Movie 3

Description: A hopper which autonomously jumps when exposed to an undesired chemical in its environment (in this case, toluene).

File Name: Supplementary Movie 4

Description: Demonstration of OR gate. Actuation of composite bistable units of PDMS-GF5 (red layers) and hydrogel-cellulose nanofibers (transparent layers) responding to toluene or water.

File Name: Supplementary Movie 5

Description: Demonstration of NAND gate. Blue units are inputs and yellow unit is output. When no unit or only one unit is exposed to toluene, the output unit remains open (i.e., $0+0\rightarrow1$, $1+0\rightarrow1$, and $0+1\rightarrow1$). When both input units are exposed to toluene, the output unit closes (i.e., $1+1\rightarrow0$).

File Name: Supplementary Movie 6

Description: A box autonomously opens when exposed to toluene, due to the actuation of a PDMS-based unit. This illustrates the simple embodied logic function **Open**($S_{toluene}$).

File Name: Supplementary Movie 7

Description: Actuation of three PDMS-based units with different w_0/L_0 (resulting in different actuation times).

File Name: Supplementary Movie 8

Description: A box with control logic **TimedOpen**($S_{toluene}$ | $\Delta t^*=20$ s), i.e., the box opens when exposed to toluene, and then closes after a defined time interval (~20 s).

File Name: Supplementary Movie 9

Description: A locked "flytrap", which requires removal of a lock (via actuation of a PDMS-GF unit) and placement of a mass in order to close. Two stimuli, $S_{toluene}$ and S_{mass}, are required, with embodied logic

Close($S_{toluene} \wedge S_{mass}$).

File Name: Supplementary Movie 10

Description: A locked "flytrap", with the lock operated by two different actuating PDMS-GF units. These are designed to actuate at two different times, with the first unlocking the flytrap, and the second re-locking it. To successfully close the flytrap, a mass must be placed on a platform during the interval between the two actuation times (while the lock is disengaged). We denote this behavior as **Close**($S_{toluene}$, S_{mass} | $t^*_{mass} - t^*_1 < t^*_2 - t^*_1$), where, in this case, $t^*_2 - t^*_1 = 10$ s. In this video, the mass is placed at the correct time, and the box closes.

File Name: Supplementary Movie 11

Description: Just like Supplementary Movie 10, the logic of this "flytrap" is **Close**($S_{toluene}$, S_{mass} | $t^*_{mass} - t^*_1 < t^*_2 - t^*_1$) with $t^*_2 - t^*_1 = 10$ s. Unlike Supplementary Movie 10, however, the mass is intentionally placed at the wrong time (after the lobe has already been relocked, via the second actuation event). As a result, the box does not close.

File Name: Supplementary Movie 12

Description: A multimaterial box system that includes one PDMS-based unit and one hydrogel-based unit. In order for the lid of the box to open, the hydrogel (triggered by water) must actuate and remove a lock from the lid. The PDMS can then open the lid if it has been exposed to a non-polar solvent. The logic is **Open**($S_{water} \wedge S_{toluene}$ | $t^*_{hydrogel} < t^*_{PDMS}$). In this movie, both water and toluene are placed on the correct actuating units. The hydrogel-based unit actuates and remove the lock first, and then the PDMS-based unit actuates to open the box.

File Name: Supplementary Movie 13

Description: The same system as in Supplementary Movie 12 (with embodied logic given by **Open**($S_{water} \wedge S_{toluene}$ | $t^*_{hydrogel} < t^*_{PDMS}$), but now intentionally incorrectly operated. Toluene is applied to both units, which actuates the PDMS unit as expected, but has no effect on the hydrogel. Without water, the hydrogel does not actuate, and, as a result, the box remains locked.

File Name: Supplementary Movie 14

Description: Self deployment of a 2D sample (PDMS-GF15 material) when exposed to toluene.
